# Perceptional gaps among women, husbands and family members about intentions for birthplace: a cross-sectional study^1^

**DOI:** 10.1590/1518-8345.1658.2840

**Published:** 2017-01-30

**Authors:** Yoko Shimpuku, Frida Elikana Madeni, Shigeko Horiuchi, Sebalda Charles Leshabari

**Affiliations:** 2 PhD, Assistant Professor, St. Luke’s International University, College of Nursing, Tokyo, Japan.; 3 MSc, RN, Magunga District Hospital, Korogwe, Tanga, Tanzania.; 4 PhD, Professor, St. Luke’s International University, College of Nursing, Tokyo, Japan.; 5 PhD, Senior Lecturer, Muhimbili University of Health and Allied Sciences, School of Nursing, Dar es Salaam, Tanzania.

**Keywords:** Skilled Nursing Facilities, Delivery, Obstetric, Midwifery

## Abstract

**Objective:**

women are more likely to give birth at a health facility when their families agree with the birthplace. However, in rural areas of Tanzania, women are often marginalized from decision-making. This study predicted birthplace intention and identified factors to reduce perceptional gaps among pregnant women, husbands and family members.

**Method:**

explanatory cross-sectional survey was conducted in three villages in North Eastern Tanzania. Participants were 138 pregnant women and their families who answered the Birth Intention Questionnaire (BIQ), measuring knowledge, attitude, perceived behavioral control, subjective norms and intention for birthplace. Descriptive analysis, ANOVA, Chi-square, and multiple linear regression was used to analyze the data.

**Results:**

the regression model showed that knowledge, perceived behavioral control, and subjective norms predicted intention for birthplace (R^2^ = .28). While 81% of pregnant women thought their husbands were decision-makers for their birth, only 38% of husbands and 37% of family members agreed. Pregnant women had significantly lower scores on the item “I will prepare for childbirth with my family” compared with husbands (p < .01) and other family members (p < .001).

**Conclusion:**

providing evidence-based birth preparation and reducing the identified perceptual gaps may enhance women’s intention to deliver at health facilities.

## Introduction

People-centered care is one of the key health policies of the World Health Organization to achieve Universal Health Coverage^1^. Therefore, more research on women’s voices needs to be conducted so that health care service meets their needs^2^. A case in point is that in rural Tanzania, skilled birth attendants (SBAs) conducted only 42.3% of all deliveries^3^ although access to SBAs in health care facilities is strongly recommended^4^. As a result, the maternal mortality ratio (MMR) per 100,000 live births of Tanzania was 410 in 2013^5^. This stalled the progress of Millennium Development Goal 5. Much effort is needed to achieve the Sustainable Development Goal 3: reducing MMR to 70.

In 2002, the Ministry of Health and Social Welfare of Tanzania introduced the “Focused Antenatal Care Package,” which pomotes delivery by SBA, preparation of childbirth and readiness for complications that may occur in pregnancy, labour, delivery and postpartum^6^. The policy was in accordance to the WHO publication of “Birth and Emergency Preparedness in Antenatal Care”^7^ that defined the nine birth preparation elements as follows: 1. the desired place of birth; 2. the preferred birth attendant; 3. the location of the closest appropriate care facility; 4. funds for birth-related and emergency expenses; 5. a birth companion; 6. support in looking after the home and children while the woman is away; 7. transport to a health facility for the birth; 8. transport in the case of an obstetric emergency and 9. identification of compatible blood donors in case of emergency. Research about these elements indicated that birth preparedness could be the key factor that influences birthplace^8^ ^-^ ^9^.

Researchers realized that birth preparedness should be discussed within the context of the families’ decision-making process. A study in Uganda found that when women prepared for birth in consultation with family members, they were more likely to give birth with SBAs, compared to women who made birth plans by themselves^10^. Another study in Tanzania also investigated partners’ influence on women’s birthplace and found that agreement of partners on the importance of delivering in a health facility was positively associated with delivery in a health facility^11^. However, several studies showed lack of decision-making power of women among their family with regard to the referral and place of birth^4^ ^,^ ^12^ ^-^ ^13^. It was also reported that husbands typically served as decision-makers about where their wives would deliver^11^ ^,^ ^14^. Therefore, even when pregnant women were aware of birth preparedness, the decision-maker of the household must have the intention for his wife to give birth at a health facility. 

The other important factors identified in the previous studies were education levels and knowledge about potential dangers during birth. Using the concept of birth preparedness, a survey was conducted in Mpwapwa district, Dodoma Region, Tanzania^15^. In their analysis, women with primary education and above and those who knew more than three danger signs were more prepared for birth and complications. 

Although the former studies^4^ ^,^ ^11^ ^-^ ^13^ identified that birth preparation and family agreement was important for safe childbirth, the findings did not explain how they had, or did not have, the intention to give birth at a facility, as they did not include the motivational factors in their studies. Although it is important to evaluate people’s intentions and related factors on birthplace, many studies simply analyzed the relationships between non-motivation factors and their behaviors. 

Ajzen’s theory of planned behavior explains that intentions are assumed to capture the motivation factors including how strongly one is willing to and how much effort one makes to perform the behavior^16^. The theory also includes non-motivational factors, such as availability of requisite opportunities and resources (e.g., time, money, skills, or cooperation of others) because these factors represent people’s perceived behavioral control (PBC). With empirical studies, the theory also explains two other conceptually independent determinants of intention: attitudes toward behavior and subjective norms (SN), which are the perceived attitudes and judgements of significant others. 

Therefore, this study examined intention for birthplace and related factors, such as knowledge, attitude, SN, and PBC among pregnant women, husbands and other family members in rural Tanzania. Our research questions included: 1. whether Knowledge, PBC, Attitude, and/or SN predicted Intention, 2. whether any of demographic items predicted Intention, and 3. were there differences among pregnant women, husbands, and family members with respect to Knowledge, PBC, Attitude, SN, and Intention. The proportions of responses were investigated to compare family members’ responses.

## Methods

This cross-sectional survey occurred in Korogwe District, located in the center of the Tanga Region of North Eastern Tanzania. The rural population was 175,339 and the urban was 43,510. The Korogwe district had one public and one private hospital, 42 dispensaries, and five health centers^17^. Medical service was considered accessable if located within 30 minutes^18^. To investigate households without regular access to medical service, we selected mountainous villages where the nearest health center was at least 5 km away, taking more than 30 minutes to walk. Lacking transporation women walked unpaved, unlit mountain roads to reach the nearest health center. Therefore, preparation of transportation and financial support was necessary to access skilled care. 

The participants of this study were pregnant women and their family members. The inclusion criteria for pregnant women were: 16 years old or older, currently pregnant with no severe physical and psychological illness. The criteria for family members were: 16 years old or older, live with or near the pregnant woman, and defined as “family” by the pregnant woman regardless of their blood or marital relationship. Women from the village did not attend the clinic on a regular basis; hence we used non-probablity sampling and village leaders to identify women who were pregnant. A female Tanzanian researcher (the initials of second author) contacted the village leader in advance to explain the study and to call for potential participants. After the research team arrived at the villages, the village leader requested the potential participants to gather at a school or a church. We explained the study and the protection of their rights to the recruited women and their families. If they agreed, a questionnaire was distributed and self-administered. A sample size of at least 100 was needed and thus recruited to meet the assumption of a normal distribution^19^. Data were collected in August 2013. 

To measure intention for birthplace and birth preparedness, the first and second authors developed the 38-item Birth Intention Questionnaire (BIQ) for pregnant women and their family members using Ajzen’s theory of planned behavior^16^. Two equivalent versions of the questionnaires were developed using “I” or “she” as appropriate for the participant. The questionnaire was first developed in English and translated into Kiswahili by the Tanzanian master’s prepared midwifery researcher (the intials of the second author) to accomodate the target population. To establish face validity and translation quality the questionnaire was pre-tested with 10 pregnant women in a similar village population. Thus several questions were revised and the questionnaire was finalized for the study. 

There were 10 knowledge items that were true/false questions about safe pregnancy and danger signs, which were derived from the Integrated Management of Pregnancy and Childbirth^20^. The higher the score, the more accurate knowledge they have. We developed nine items to tap into the concept of perceived behavioral control (PBC), which refers to the perceived ease or difficulty of performing the behavior and it is assumed to reflect past experience as well as anticipated impediments and obstacles^15^. We asked if they had felt ease or difficulty: saving money for birth, receiving family support, identifyingtrained health care provider, finding a health care facility, reaching the health care facility, finding transportation, and reading family agreement for health care. These seven items were scored on a three-point Likert scale; agree (3 points), neither agree nor disagree (2 points), or disagree (1 point). The Cronbach’s alpha was 0.662. 

Attitude toward behavior refers to the degree to which a person has a favorable or unfavorable evaluation or appraisal of the behavior in question^16^. The four items asked if they prefered:, home birth or facility birth, birth with traditional birth attendants, birth with skilled birth attendants, avoiding health center due to cesarsean-section or episiotomy. The Cronbach’s alpha was 0.657.

Subjective norms (SN) are six items about perceived social pressure to perform or not to perform the behavior: social pressure about working during pregnancy, obedience to husband’s intention, taking care of children at birth, and submission to family’s wishes. The Cronbach’s alpha was 0.652.

Intention for birthplace includes seven items about motivation to achieve their plan regarding pregnancy and childbirth: have healthy diet for pregnancy, go to health facility for danger signs, prepare for birth with family, give birth at health care facility, and go to health center for postpartum problem. The Cronbach’s alpha was 0.553.

The socio-demographic items included: age, education, occupation, financial capacity, household assets, ethnic group, and experience of loss in pregnancy or childbirth. Also, the last two questions asked who decides where to give birth, and who they want to be with when they give birth. 

SPSS ver. 22.0 was used for data analysis. Descriptive analysis was used to illustrate the proportions of responses; ANOVA, Chi-square, or Fisher’s exact test were used to investigate statistical differences among and between the groups. Multiple regression analysis was conducted to identify which variables predicted Intention for birthplace. Correlations were also analyzed to identify relationships between variables. For variable scores, missing data were filled using the mean score.

We obtained oral consent because the information gathered was unidentifiable and the risk from this study was minimal. Ethical clearance and permission were obtained from 1. the Resaerch Ethics Committee of St. Luke’s International University, 2. Director of Korogwe District Council, 3. the National Institute for Medical Research (NIMR) and 4) the Tanzania Commission for Science and Technology (COSTECH). 

## Results

Responding from the three villages was a total of 139 participants. All chose to participate in the survey; however, for reasons unknown, one participant did not complete the questionnaire and therefore was excluded. Hence, 138 participants, 42 pregnant women, 35 husbands and 44 family members (seven were mothers of the husbands and three were mothers of the women) were included in the analysis. Table 1 shows the socio demographic characteristics. Although there was statistically significance in age and household assets, they did not affect other statistical differences as covariates.

**Table 1:** Comparison of socio-demographic characteristics of pregnant women, husbands and family members. Tanzania, 2013

Characteristics		Pregnant women	Husbands	Family members	*p*-value
Total (n)		42	35	44	
Age (mean)		27.67	33.21	36.41	0.008*
Education level					0.089
	Secondary and above (%)	17	11.8	38.5	
	Primary or less (%)	82.9	88.2	61.5	
Occupation					0.157
	Farmer (%)	80.5	88.2	60.0	
	Housewife/student (%)	17	0	25.0	
	Others (%)	2.4	11.8	15.0	
Daily expense					0.346
	<1000 TSH (%)^†^	42.9	54.9	41.5	
	1000-5000 TSH (%)	50	35.5	39	
	≥5000 TSH (%)	7.1	9.7	19.5	
Household assets ownership					0.028^‡^
	Low (0-1) (%)	90.5	74.3	75.0	
	High (2+) (%)	9.5	25.7	25.0	

* p < 0.05; ^†^ TSH: Tanzania Shillings; ^‡^ p < 0.01

**Figure 1:**
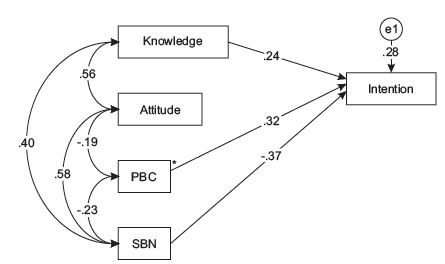
Conceptualization of Birth Intention Questionnaire

For the research question 1., whether Knowledge, PBC, Attitude, and/or SN predict Intention, the regression model (Figure 1) showed that Knowledge, PBC, and SN predict Intention (R^2^ = 0.28). SN had a negative influence on Intention. Attitude was dropped from the regression model; however, Attitude was correlated with Knowledge and SN (*r* = 0.56, 0.58, respectively, *p* < 0.001). Research question 2., asked whether any of demographic items predict Intention; all the demographic items were dropped as a result of multiple linear regression.

* PBC: Perceived Behabioral Control; † SBN: Sobjective Norm

For the research question 3., there were no significant differences among pregnant women, husbands, and family members with respect to knowledge, PBC, attitude, SN, and Intention. Despite the statistical non-significance, there were several useful findings at the item level as follows. 

**Figure 2:**
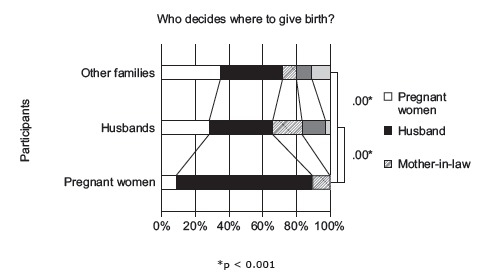
Decision makers for birthplace

A significant majority of pregnant women (81.1%) (*p* < 0.001) responded the husband decides where to give birth, whereas about 37 % husbands and family members answered the husband was the decision-maker. Conversly, 27.6% of husbands and 34.3% of family members thought that pregnant women decide where to give birth; yet only 8.1% of pregnant women answered they decided their birth place (Figure 2).

A majority (65.9%) of pregnant women answered that they want to be with either a doctor or nurse during childbirth, whereas 46.7% of husbands and 55.9% of family members chose either a doctor or nurse. 

Pregnant women had significantly (*p* < 0.05) higher scores compared to husbands and family members about the use of traditional medicine. They scored significantly less than husband and family about the use of iron tablets (*p* < 0.05). 

Regarding perceived behavior control for saving money for childbirth, agreeing were 76.7% of family members, 62.9% of husbands, and only 50% of pregnant women. In contrast, women tended to show more perceived ease of preparation for facility-based delivery. The majority of participants agreed there was a family member to accompany the woman to the health facility for birth. 

For Attitude about half of participants had a reluctance to use facility-based care because of a negative perception toward medical intervention although more women prefered facility-based delivery, compared to husbands and family members. A minority of all respondents felt that traditional birth attendants were kinder than nurses at the hospital. 

For Intention the biggest discrepancy was, “I will prepare for childbirth with my family (or with her [pregnant women]).” While only 32.5% of pregnant women agreed, 61.8% of husbands and 81.4% of family members agreed resulting in significant differences between pregnant women and husbands (*p* < 0.001) as well as between pregnant women and family members (*p* < 0.01). 

## Discussion

In this survey of pregnant women, husbands, and family members occurring in the rural mountainous villages in Korogwe District, the regression model identified that Knowledge and PBC predicted Intention. In contrast to the theory, SN negatively predicted Intention. As SN items asked if they chose their own safe delivery based on what is recommended in the Western world, rather than loyalty to family, the theory might not have fully captured the Tanzanian subjective norms. The successful communication negotiation strategies may differ from the Western world^11^.

The family decision-making process in rural Tanzania might explain the following discrepancy; a large majority of women thought husbands decided the birthplace, whereas only a minority of husbands thought of themselves as decision-makers. A qualitative study in the coastal region of Rufiji depicted that when health workers recommended the referral, the advice was transmitted by the woman to her husband and the woman or husband consulted with mother-in-law, woman’s parents, and several family members^12^. Usually the husband was the final decision-maker and responsible to finance the transport; however, his decision was often influenced by other family members. 

Another important finding is that women avoided showing their intention for their own childbirth or being part of decision-making and preparation. The majority of women did not agree with “I will prepare for [*Tutaandaa*] childbirth with my family.” The Kiswahili expression of *Tutaandaa* could be translated to “We will prepare” and indicates that women are part of decision-making and preparation. With consideration of women’s relatively high knowledge scores and preference of SBAs in this study, it is likely that women understood the possible danger of childbirth and preferred skilled care; however, loyalty to the family might hinder her from showing her intention and describing that she is part of decision-makers. 

The issue of reluctance to declare their intention could be related to their diminished financial capacity. Women’s household assets ownership was significantly lower than husbands and other family members. Only half of pregnant women in this study agreed that they had saved money for a facility birth. This is lower than the findings of the study in central Tanzania^15^, in which 89% of women reported saving money in case of emergency. This may reflect the marginal economic capacity of rural women in this population. It is plausible that the perceived ease or difficulty of women performing the behavior was increased by the amount of money women could use, or felt entitled to use^12^. Another study in Ethiopia showed a close relationship between women’s autonomy (or position in the household) and maternal healthcare utilization^21^. The authors indicated the importance of promoting income-generating activities and education among women so that women’s position in the household is enhanced.

Yet, it is important to notice that husbands and family members in this study thought that women were the decision-makers more than women themselves even though the gender literature indicates that women often encourage younger women to be submissive to their husbands^22^. A study conducted in southern Tanzania reported that the pattern of decision-making power within the household was the key determinant of birthplace, and that women who lived in male-headed households were less likely to deliver in a health facility than women in female-headed households^4^. 

Because many of family agreed that women decided their own birthplace, better communication may enhance women’s willingness to speak up within the family about their intention. In this study, however, husbands and family members tended to have lower scores when it was related to the needs of support when going to a health center for deliveries; husband’s permission is necessary to go to health center; and women stay at home to take care of the children. A researcher found that if women in nuclear families had few difficulties with their husbands, they were more likely to give birth at a health facility; if women in a joint family had very few difficulties with in-laws, they were more likely to attend antenatal care^23^. As the supportive relationships with husbands and other family members develop, women could easily discuss or show their intention, which eventually benefit their health. Discussion among family members should be promoted to dissolve the perceptional gaps identified in this study. 

## Limitations 

Some differences may not have been detected due to the small sample size. However, with this relatively small sample size, we found several statistically significant relationships among the variables. In addition, the participants were recruited through non-probability sampling, and therefore they might not represent the community population. Further study is necessary with bigger and random sample so that it will detect more variation. The psychometric properties of the BIQ require further development. The BIQ used a three-point Likert scale diminishing the ability to detect the statistical difference. Despite these caveats the results provide useful direction for exploring and improving communication among families for choosing place of birth.

## Conclusion

The discrepancies among pregnant women and their families regarding intention for childbirth suggest more supportive relationships are necessary for women to voice their intention. Discussion among family members should be encouraged to fill the perceptual gaps and achieve universal coverage of skilled attendants for childbirth.
